# Myo-Inositol in the Treatment of Teenagers Affected by PCOS

**DOI:** 10.1155/2016/1473612

**Published:** 2016-08-18

**Authors:** Lali Pkhaladze, Ludmila Barbakadze, Nana Kvashilava

**Affiliations:** Archil Khomasuridze Institute of Reproductology, 0112 Tbilisi, Georgia

## Abstract

*Objective*. To compare the effectiveness of myo-inositol (MI) and oral contraceptive pills (OCPs) in monotherapy and MI in combination with OCPs in the treatment of teenagers affected by polycystic ovary syndrome (PCOS).* Methods*. 61 adolescent girls aged 13–19 years, with PCOS, were involved in the prospective, open-label study. Patients were randomized into three groups: I group, 20 patients receiving drospirenone 3 mg/ethinyl estradiol 30 *μ*g; II group, 20 patients receiving 4 g myo-inositol plus 400 mg folic acid; III group, 21 patients receiving both medications.* Results*. After receiving MI significant reduction in weight, BMI, glucose, C-peptide, insulin, HOMA-IR, FT, and LH was detected. The levels of SHBG, TT, FAI, DHEA-S, and AMH did not change statistically significantly. After receiving OCPs weight and BMI slightly increased, but metabolic parameters did not change. Combination of MI and OCPs did not change weight and BMI, but reduction in C-peptide, insulin, and HOMA-IR was detected. TT, FT, FAI, DHEA-S, LH, and AMH levels decreased and SHBG increased.* Conclusions*. Administration of MI is a safe and effective method to prevent and correct metabolic disorders in teenagers affected by PCOS. With combination of MI and OCPs antiandrogenic effects are enhanced, negative impact of OCPs on weight gain is balanced, and metabolic profile is improved.

## 1. Introduction

Polycystic ovary syndrome (PCOS) is considered to be the most common endocrine disorder in the women of reproductive age, with onset manifesting as early as puberty [1]. PCOS prevalence is estimated to be about 6–8%, although with the implementation of the Rotterdam criteria, the prevalence increased up to 15–25%, while the use of AES recommendations put PCOS prevalence at about 10–15% [2].

PCOS is a genetically determined lifelong disease. Symptoms start from the early prepubertal years and continue after menopause. The phenotypic expression varies through time, depending on several internal and external factors [3, 4].

The data suggests that exposure of a female to harmful events during fetal life and the peripubertal period may considerably affect her metabolic, hormonal, and reproductive phenotype. Dysregulation of cytochrome P450 p17, the androgen-forming enzyme in both adrenal glands and the ovaries, is the central pathogenic mechanism underlying hyperandrogenism in PCOS [5]. The presence of hyperandrogenism reduces the hepatic synthesis of SHBG and leads to a relative excess of free circulating androgens [6].

Adolescents with PCOS tend to be troubled by the cosmetic effects, such as acne, hirsutism, acanthosis nigricans, and obesity. They occur during a particularly vulnerable stage of their psychological development [7].

Insulin resistance and compensatory hyperinsulinemia are observable in at least 45–65% of PCOS patients and frequently appear to be related to excessive serine phosphorylation of the insulin receptor [5].

Patients with PCOS have an increased risk for developing type 2 diabetes, metabolic syndrome, coronary heart disease, and endometrial cancer [8, 9]. Obese PCOS adolescents are at increased risk for developing glucose intolerance and type 2 diabetes compared with their non-PCOS peers [10–12].

The preferred effective method of treatment for obese adolescents with PCOS is lifestyle modification; however it is hard for patients to comply with and achieve this [13].

Combined oral contraceptive pills (OCPs) have been used in the women with PCOS for treatment of menstrual disorders, acne, and hirsutism. Despite years of using them and broad clinical experience, there still are some ongoing doubts concerning their implications for the cardiovascular system and carbohydrate metabolism [14]. Most pediatric endocrinologists prefer to use OCPs with low androgenic potential [15].

More recently, insulin-sensitizing drugs have been proposed as another long-term treatment for PCOS. Pharmacologic reduction in insulin levels by either metformin or thiazolidinediones ameliorates both hyperinsulinemia and hyperandrogenism [16, 17]. A meta-analysis of the published studies demonstrated that the use of insulin sensitizers does not reduce hyperandrogenism any better than OCPs [18].

Adolescent girls with PCOS require a long-term committed treatment that can have serious side effects. Therefore, alternative treatments may be possible options for adolescents affected by PCOS [7].

In the last decade, a higher attention has been paid to the role of inositol-phosphoglycan (IPC) mediators of insulin action [19]. A deficiency of myo-inositol (MI) has been found in women with PCOS affected by insulin resistance [20]. The insulin resistance at the base of PCOS leads to hyperinsulinemia, which at ovarian level is responsible for the alteration in the inositols metabolism. PCOS patients with hyperinsulinemia present an enhanced MI to D-chiro-inositol (DCI) epimerization in the ovary; this results in an increased DCI/MI ratio (i.e., overproduction of DCI). A sequent reduction of MI increases the isoform DCI and induces overproduction of androgens [21–23]. MI is a component of the vitamin B complex and insulin sensitizer, which improves insulin signaling, reduces serum insulin, and decreases serum testosterone, thereby restoring normal ovulatory function in PCOS women [24–27].

Safe and effective treatment in teenagers affected by PCOS is essential for ameliorating clinical manifestations, restoring self-esteem, and preventing further complications. There are limited studies concerning usage of MI in adolescents. Therefore, the aim of our study was to compare the effectiveness of MI and OCPs in monotherapy and combination of MI and OCPs in the treatment of teenagers with PCOS by evaluation of hormonal and metabolic profile.

## 2. Materials and Methods

61 adolescent girls aged 13–19 years affected by PCOS were involved in the prospective, open-label study. The diagnosis was confirmed according to the Rotterdam criteria [28]. Patients within two years of menarche were excluded from the study. All the patients were from Archil Khomasuridze Institute of Reproductology. Informed written consent was obtained from all participants and/or their mothers (guardians) before entering the trial and the local committee of ethics approved the given study.

Healthy life style, including reduced carbohydrate intake and gentle exercise, was recommended to all patients as a 1st line nonpharmacological management, especially to overweight ones. Patients were randomly allocated into three following treatment groups: I group, 20 patients receiving monophasic low-dose combined OCPs, Yarina (drospirenone 3 mg/ethinyl estradiol 30 *μ*g, Bayer Health Care Pharmaceuticals), consumed in the evening in a cyclic regimen (from the 3rd to the 5th day of menstruation, 21 days) for 3 months; II group, 20 patients receiving Inofolic (2 g myo-inositol plus 200 mg folic acid, Lo.Li., Pharma S.r.l., Rome, Italy) consuming 2 g in the morning and 2 g in the evening for 3 months; III group, 21 patients receiving combination of Yarina and Inofolic in the same regimen for 3 months.

Evaluations of anthropometric, endocrine, and metabolic parameters were conducted before and after 3 months of treatment. Body mass index (BMI) was calculated as weight (kg) divided by height (m^2^).

Serum concentrations of prolactin (PRL), luteinizing hormone (LH), total testosterone (TT), sex hormone-binding globulin (SHBG), free-testosterone (FT), dehydroepiandrosterone-sulfate (DHEA-S), anti-Mullerian hormone (AMH), C-peptide, fasting glucose, and fasting insulin levels were assessed within the first 5 days of the menstrual cycle. Insulin resistance was measured by homeostasis model assessment (HOMA-IR) and free androgen index (FAI) was calculated by formula: ((TT nmol/l)/(SHBG nmol/l)) × 100.

Statistical analysis was performed by using SPSS (Statistical Package for Social Sciences, version 21, Chicago, USA). Data are given as mean ± SD. The baseline characteristics of the patients between groups and changes in parameters within groups after 3 months of treatment were assessed using unpaired *t*-test (ANOVA). The results were considered as statistically significant when the *p* value was less than 0.05 (*p* < 0.05).

## 3. Results

In the I group (Yarina) average age of the patients was 15.95 ± 1.85, average weight was 61.1 ± 9.92 kg, and average BMI was 22.74 ± 3.75 kg/m^2^. Among them 5 patients (25%) had BMI > 25.5 kg/m^2^. In the II group (Inofolic) average age of the patients was 16.75 ± 2.0, average weight was 58.6 ± 9.3 kg, and average BMI was 22.3 ± 3.08 kg/m^2^ and among them 4 patients (20%) had BMI > 25.5 kg/m^2^. In the III group (Yarina plus Inofolic) average age of the patients was 16.24 ± 1.86, average weight was 58.95 ± 9.6, and average BMI was 22.24 ± 3.26. Among these patients 4 (19%) had BMI > 25.5 kg/m^2^.

Hormonal and metabolic parameters of patients are listed in Table 1. Groups were well matched at baseline—there were no statistically significant differences between the groups in terms of clinical, anthropometric, hormonal, and metabolic parameters. Average LH level only was higher in III group compared with other groups (*p* < 0.05).

In the I group after receiving Yarina, patient's weight and BMI increased statistically significantly, while in the II group after receiving Inofolic, patient's weight and BMI decreased statistically significantly. In the III group, in patients treated with combination of Yarina and Inofolic, we did not reveal statistically significant changes in average weight and BMI.

The average level of PRL did not change before and after treatment in groups. The average level of LH was statistically significantly decreased after treatment in all groups, but less significantly in patients treated with Inofolic. The average level of DHEA-S did not change in patients after receiving Inofolic but decreased statistically significantly in the Yarina and combined groups.

Reduction in TT was observed in the I and III groups, but statistically significantly, only in the III group. The change was not revealed after receiving Inofolic only. However, average FT decreased statistically significantly in all groups and more significantly in the combined group. Average SHBG level was increased in all treatment groups, but statistically significantly only in the I and III groups. Accordingly, FAI was reduced significantly in the Yarina and combined groups and change was not detected in the Inofolic group.

The average level of AMH statistically significantly decreased in patients receiving Yarina only and combination of Yarina and Inofolic, but more significantly in I group. But in the II group, after treatment of Inofolic only, it increased, but not statistically significantly.

Before treatment all patients had normal levels of fasting glucose, but hyperinsulinemia and insulin resistance were observed in 6 (30%) and 5 (25%) patients in I group, in 6 (30%) and 4 (20%) patients in II group, and in 6 (29%) and 5 (24%) patients in III group, respectively.

Statistically significant reduction in average of glucose, C-peptide, insulin concentration, and HOMA-IR was detected in patients after receiving Inofolic. However, in the patients receiving Yarina only statistically significant changes were not revealed in average concentrations of C-peptide, insulin, and HOMA-IR. However, the average levels of glucose decreased statistically significantly. All these parameters significantly decreased in patients treated with combination of Yarina and Inofolic, except for the glucose concentration; the change was not statistically significant.

## 4. Discussion

PCOS is quite a complex syndrome and should not be considered as “an easy to treat” disease. It needs a precise clinical screening that might give suggestions on what hormonal and metabolic parameters need to be treated [29]. This issue is extremely important in adolescents, due to the diagnostic difficulties in this period.

In the first year after menarche half of the cycles are anovulatory in healthy adolescents. If menstrual irregularities persist 2 years after menarche the risk of PCOS is extremely high, 70% of cases [30].

Generally, the first line nonpharmacological management of PCOS includes life style modification with loss of weight. It has been proven as an effective way for restoring ovulatory cycles and achieving pregnancy in overweight women with PCOS [31]. Therefore, healthy life style modification was recommended to all our patients, with reduced carbohydrate intake and gentle exercising to those who were overweight.

Long-term management of PCOS most often involves the use of OCPs [32]. OCPs effectively reduce hirsutism and acne in women with PCOS. This improvement is usually revealed in 60–100% of cases after at least 6 months of treatment [33]. The effect of OCPs on hyperandrogenism includes suppression of androgen production in the ovary by inhibiting secretion of LH, induction of SHBG synthesis in the liver and consequent decrease in FT, slight decrease in adrenal androgen production, and direct antiandrogenic effect of a progestin component of OCPS [34]. According to the results of our study after receiving Yarina TT decreased, but not statistically significantly, which can be explained by small size of the group. However, increase in SHBG and decrease in FAI and FT were detected. DHEA-S decreased as well. We did not reveal reduction in androgens after treatment with MI in our study in contrast to others [35]. It can be related to limited period of our observation. However, after treatment with MI decrease of LH level was detected, which is a precondition for antiandrogenic activity and is reported by other authors as well [35]. It should be emphasized that after receiving MI the amount of FT is reduced.

With combination of MI and OCPs antiandrogenic activity was extremely marked and was expressed by significant reduction in TT, FT, DHEA-S, FAI, and LH.

Recent studies demonstrate effectiveness of MI in the treatment of hirsutism and other cutaneous disorders in young women with PCOS [35, 36]. Due to the short duration of our study, we did not evaluate the expression of clinical manifestations, like hirsutism, acne, acanthosis nigricans, and changes in menstrual cycle, but positive effect of all medications on hormonal profile is apparent.

The serum AMH level strongly correlates with the number of small antral follicles and is closely related to the degree of menstrual disturbances [37]. Therefore high AMH levels were observed in patients with PCOS. This is particularly useful for the diagnosis of PCO [38, 39]. In our study baseline level of AMH was elevated almost in all patients. After treatment with OCPs only and in combination with MI, AMH level decreased, while after treatment with MI only trend to increase AMH was detected.

MI improves response to clomiphene citrate in infertile women, restores ovulation, and increases clinical pregnancy and live birth rate [40]. Latest studies proved effectiveness of MI in improving quality of oocytes in IVF cycles [41]. Therefore, these beneficial effects of MI would be helpful in adolescents with PCOS to prevent reproductive disorders in future.

Typically, after receiving OCPs weight increases. In drospirenone containing pills, this effect is less expressed [42]. In our study after receiving Yarina average weight and BMI slightly increased. It could be related to increase of appetite.

We consider weight loss and BMI decreasing to be quite pivotal after receiving MI. In the study of Gerli et al. a significant weight loss in patients treated with MI was recorded [43]. In other studies changes in BMI of reproductive age women were not detected [35]. We underline that in our study in patients treated with combination of MI and OCPs average weight and BMI did not change. Apparently, MI balances negative impact of OCPs.

The studies conducted in obese women with PCOS demonstrated a deterioration of glucose tolerance with OCPs administration likely due to a decrease in insulin sensitivity with no change in plasma insulin concentrations [44, 45]. This occurred despite no change in BMI and a marked decrease in circulating androgens. Studies performed in nonobese women with PCOS showed no change in glucose tolerance or insulin sensitivity after OCPs, suggesting that the metabolic effects of OCPs may vary with body phenotype [46, 47]. During usage of drospirenone the metabolic effects appear to be much less severe or entirely nonexistent when women with PCOS are treated with drospirenone containing OCPs. Guido and authors found no significant change in insulin sensitivity in PCOS women treated with drospirenone containing OCPs [48]. The use of drospirenone appears to alleviate the metabolic concerns that are specific for women with PCOS [15].

In our study MI showed good results in terms of controlling metabolic parameters, glucose, C-peptide, insulin, and HOMA-IR, and demonstrated slight antiandrogenic activity, which is reported by other authors as well [43]. According to our results, changes in metabolic parameters matched with weight reduction. It is very important to underline that during receiving Yarina metabolic profile did not change, but in combination of MI, it improved. It is considerable finding that MI can prevent developing of serious metabolic disturbances in adolescents with PCOS in future.

According to the literature, minimal weight loss of 2–7% of body weight reduces androgen levels and improves ovulatory function in many patients with PCOS [49]. In adolescents lifestyle modification can only result in a 59% reduction of free androgen index with a 122% increase in SHBG [50]. So, it is difficult to say how the modification of lifestyle and administration of medications contributed to reduction of androgens and controlling metabolic parameters in our study.

## 5. Conclusion

Administration of MI is a safe and effective method to prevent and correct metabolic disorders in teenagers affected by PCOS. With combination of MI and OCPs antiandrogenic effects are enhanced, negative impact of OCPs on weight gain is balanced, and metabolic profile is improved.

A simultaneous treatment with MI and OCPs (containing drospirenone) on the basis of life-style modification can be considered as a highly effective approach in teenagers affected by PCOS.

## Figures and Tables

**Table 1:** Anthropometric, endocrine, and metabolic parameters of the patients in treatment groups.

Parameter M ± SD	Yarina		Inofolic		Yarina + inofolic	
	Group I		Group II		Group III	
	Baseline	After 3 months	Baseline	After 3 months	Baseline	After 3 months
BMI kg/m^2^	22.74 ± 3.75	23.03 ± 3.6^*∗*^	22.3 ± 3.08	21.9 ± 2.5^*∗*^	22.24 ± 3.26	21.99 ± 2.57
Weight kg	61.1 ± 9.92	61.9 ± 9.47^*∗*^	58.6 ± 9.3	57.4 ± 7.6^*∗*^	58.95 ± 9.6	58.05 ± 7.6
PRL ng/ml	11.70 ± 5.11	12.01 ± 4.5	12.98 ± 5.75	12.5 ± 5.32	13.39 ± 4.5	13.03 ± 4.35
LH IU/I	10.42 ± 3.9	8.01 ± 2.9^*∗∗*^	9.11 ± 5.7	7.56 ± 4.5^*∗*^	13.1 ± 5.58	8.42 ± 3.7^*∗∗*^
DHEA-S *µ*g/ml	2.03 ± 0.7	1.82 ± 0.46^*∗*^	2.09 ± 0.50	1.89 ± 0.59	2.3 ± 0.67	1.9 ± 0.38^*∗*^
TT ng/ml	0.72 ± 0.25	0.58 ± 0.57	0.73 ± 0.25	0.73 ± 0.46	0.70 ± 0.23	0.51 ± 0.16^*∗∗*^
FT pg/ml	4.26 ± 2.46	2.74 ± 1.16^*∗*^	3.82 ± 2.01	2.98 ± 0.94^*∗*^	3.42 ± 2.16	2.12 ± 0.8^*∗∗*^
FAI	7.13 ± 3.8	3.8 ± 2.6^*∗∗*^	6.7 ± 5.7	5.99 ± 5.24	8.1 ± 5.5	2.94 ± 1.04^*∗∗*^
SHBG nmol/l	45.03 ± 24.39	55.42 ± 25.7^*∗∗*^	53.7 ± 27.4	56.29 ± 24.3	47.4 ± 29.2	65.01 ± 22.5^*∗∗*^
Glucose mmol/l	4.61 ± 0.74	4.36 ± 0.53^*∗*^	4.64 ± 0.65	4.39 ± 0.56^*∗*^	4.64 ± 0.72	4.42 ± 0.52
C-peptide ng/ml	1.6 ± 0.7	1.53 ± 0.34	1.92 ± 0.7	1.82 ± 0.34^*∗*^	1.52 ± 0.7	1.22 ± 0.57^*∗*^
Insulin *µ*IU/ml	8.07 ± 5.24	8.12 ± 4.3	8.5 ± 6.7	5.2 ± 3.04^*∗*^	8.67 ± 4.7	6.67 ± 2.79^*∗*^
HOMA-IR	1.59 ± 1.07	1.57 ± 0.87	1.81 ± 1.38	1.03 ± 0.64^*∗*^	1.73 ± 1.0	1.3 ± 0.56^*∗*^
AMH ng/ml	11.72 ± 5.8	10.26 ± 4.6^*∗∗*^	11.5 ± 5.8	12.6 ± 6.25	12.01 ± 5.6	10.4 ± 4.7^*∗*^

Comparison of the data at baseline and after treatment within the groups. ^*∗*^ *p* < 0.05; ^*∗∗*^ *p* < 0.001.
